# Case report: Lichenoid esophagitis revealing an HIV infection

**DOI:** 10.3389/fmed.2024.1477787

**Published:** 2024-10-23

**Authors:** Jasmin Marschner, Annette Schmitt-Graeff, Wolfgang Kreisel, Annegrit Decker, Franziska Schauer

**Affiliations:** ^1^Department of Dermatology, Medical Center-University of Freiburg, Faculty of Medicine, Freiburg, Germany; ^2^Medical Center-University of Freiburg, Faculty of Medicine, Freiburg, Germany; ^3^Department of Medicine II, Gastroenterology, Hepatology, Endocrinology and Infectious Diseases, Medical Center-University of Freiburg, Faculty of Medicine, Freiburg, Germany

**Keywords:** lichen planus, stroke, HIV, esophagitis, dysphagia, Epstein–Barr virus, penile cancer

## Abstract

Esophageal lichen planus is an underrecognized manifestation of lichen planus. It is typically diagnosed based on characteristic endoscopic findings, such as hyperkeratosis, trachealization, denudation and/or stenosis, along with the presence of a lichenoid infiltrate in histopathological examination. In cases where no other manifestation of lichen planus are found and direct immunofluorescence for fibrinogen along the basement membrane is negative, the term “lichenoid esophagitis” should be preferred. This distinction is critical, as it prompts a thorough evaluation for underlying diseases, including autoimmune conditions and viral infections. We report a case of a 69-year-old male with stenosing esophagitis resembling esophageal lichen planus on endoscopic evaluation. The condition was refractory to multiple dilation procedures and high-dose proton pump inhibitor therapy. Histopathological analysis revealed a dense lymphocytic infiltrate extending into the epithelial layer, while direct immunofluorescence microscopy for fibrinogen was negative. There were no other signs of lichen planus on the skin or mucous membranes. The patient’s medical history included recurrent transient ischemic attack (non-cardiac), penile cancer and recurrent mucosal candidiasis. Laboratory findings revealed Epstein–Barr virus viremia and IgG hypergammaglobulinemia, raising suspicion of immunodeficiency. Further testing confirmed an active HIV infection, classified as category C3, and antiretroviral therapy was initiated. Following the initiation of antiretroviral therapy, the patient experienced rapid clinical and histopathological improvement of the lichenoid esophageal inflammation, although the esophageal stenosis persisted. Subsequent follow-up endoscopies confirmed resolution of the inflammatory component, underscoring the positive impact of addressing the underlying HIV infection on the esophagus. This case report highlights the importance of recognizing lichenoid esophagitis as a potential diagnosis in cases of unexplained chronic esophagitis, especially when standard treatments are ineffective. The presence of lichenoid inflammation without other manifestations of lichen planus should trigger an investigation into underlying conditions.

## 1 Introduction

### 1.1 Background on lichen planus

Lichen planus is a common chronic inflammatory disease that affects the skin, mucous membranes and skin appendages (1). This condition is known for its distinctive appearance and can significantly impact the quality of life for those affected.

### 1.2 Esophageal lichen planus

Esophageal lichen planus is rare and often underdiagnosed due to its similarity to other forms of esophagitis (2–4). Accurate diagnosis typically requires endoscopic evaluation and histological confirmation. However, its actual prevalence remains elusive, largely due to frequent underdiagnosis (2, 4).

### 1.3 Challenges in diagnosing esophageal lichen planus

Esophageal lichen planus can easily be misdiagnosed as another form of esophagitis. The challenge in diagnosing esophageal lichen planus stems from several factors: it may not coincide with oral or cutaneous lichen planus manifestations, its milder forms can be asymptomatic and definitive diagnosis necessitates endoscopic evaluation (3, 5).

### 1.4 Endoscopic and histological features

Both endoscopically and histologically esophageal lichen planus shares features with other forms of esophagitis, complicating differential diagnosis (6). In esophageal lichen planus endoscopy findings are hyperkeratosis, trachealization and most importantly subepithelial denudation (3, 5–7). Histologically, it is characterized by a lymphocytic band-like infiltrate in the superficial lamina propria, presence of apoptotic keratinocytes (Civatte bodies), varying degrees of epithelial detachment from the lamina propria, and epidermoid metaplasia (2, 5). Direct immunofluorescence (DIF) microscopy can be helpful to reinforce the diagnosis detecting fibrinogen, globular IgM and/or complement deposition at the epithelial basement membrane (5). In case of a negative DIF or an absence of cutaneous lichen planus some authors prefer to label the pathology as lichenoid esophagitis pattern (2). Both entities have the potential to cause long-term esophageal strictures (3).

### 1.5 Importance of diagnosis in systemic conditions

Here we present the case of a patient with chronic erosive esophagitis, lacking cutaneous or other mucous membrane involvement, eventually diagnosed with lichenoid esophagitis. This diagnosis was pivotal in uncovering an undetected HIV infection, illustrating the complex interplay between esophageal pathology and systemic conditions.

## 2 Case description

**Figure 1:**
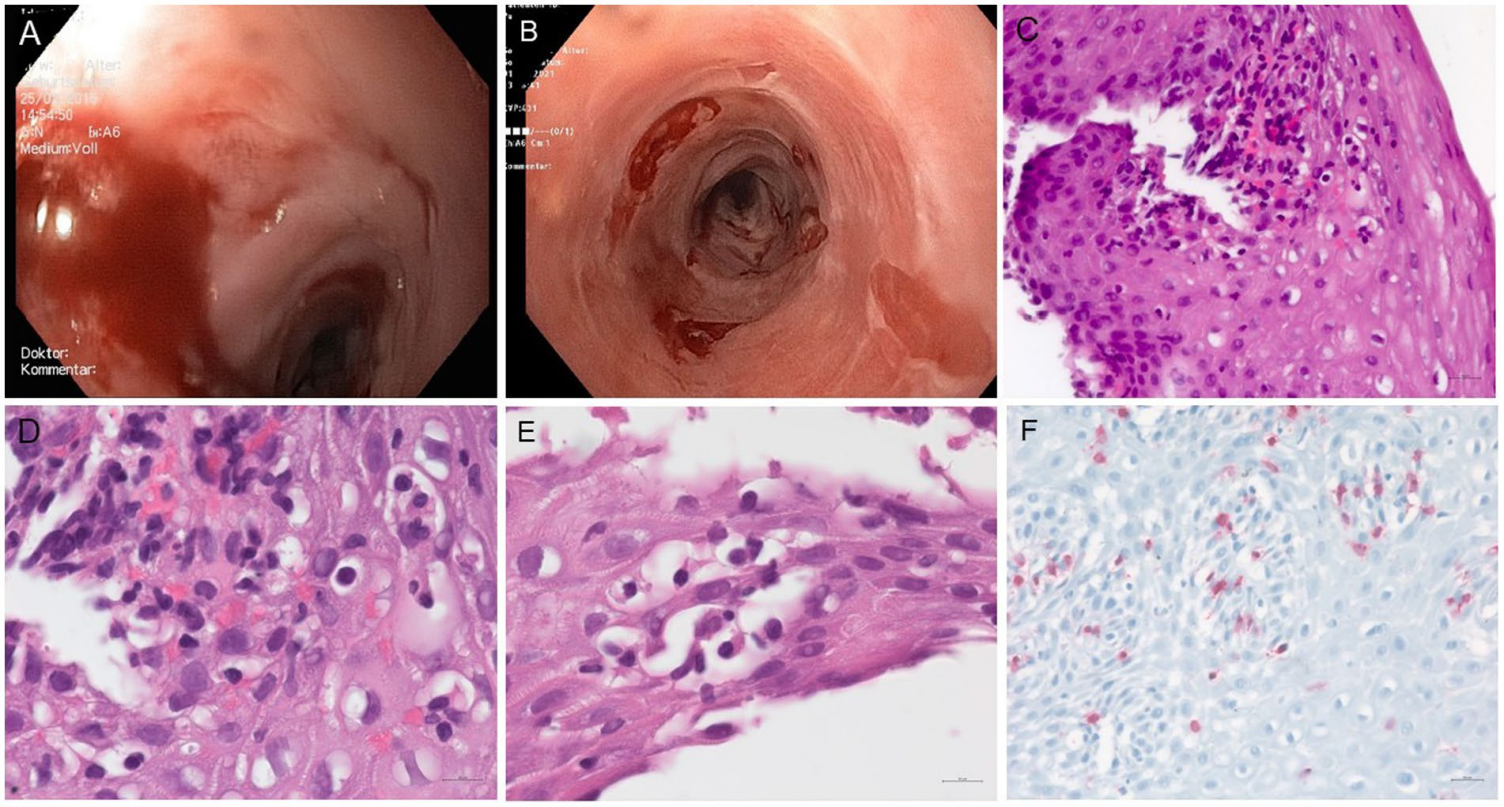
**(A)** Endoscopy of the patient’s esophagus in 2015: the mucosa of the middle esophagus is highly prone to contact vulnerability, experiencing partial mucosal detachment during device passage, with several scarring strictures located 39 cm from the dental arch (not shown). **(B)** Esophageal endoscopy conducted at the time of HIV diagnosis in 2021 revealed mild trachealization in the middle and lower thirds of the esophagus, along with areas of scarring. Iatrogenic denudation occurred after esophageal biopsies. Additionally, there is a ten millimeter stenosis located 35 cm from the dental arch (not shown). **(C–F)** Microscopic features of the patient’s esophageal biopsies: **(C–E)** H&E stained sections show detached squamous epithelium with marked spongiosis and predominantly lymphocytic inflammatory infiltrates. **(F)** Immunolabelling for CD3 highlights the presence of numerous intraepithelial T-cells (original magnification C × 40, D, E × 65, F × 25).

This is the report of a 69-year-old European male who underwent extensive treatment for erosive and stenosing esophagitis (Figure 1A) at our gastroenterology department over several years. Previous differential diagnoses, such as eosinophilic or lymphocytic esophagitis, were ruled out. Chronic antral gastritis, consistently observed during endoscopies, and suspected reflux esophagitis, led to the administration of proton pump inhibitors (PPI). Sporadic dilations allowed transient improvement of food intake for pureed foods. Empirical therapies using topical glucocorticosteroid were without longstanding effect and led to candida infections of the oral and esophageal mucosa. Despite these interventions, only transient symptomatic relief was achieved, primarily facilitating the intake of pureed foods.

Over the years, the patient’s condition progressively worsened, evidenced by weight loss, persistent dysphagia, and fatigue. Additionally, he experienced recurrent transient ischemic attacks with no discernible cardiac etiology. Additionally, he was diagnosed with a penile squamous cell carcinoma, moderately differentiated and HPV16 positive, with an isolated lymph node metastasis which was treated curatively.

Endoscopic examination of the esophagus in 2021 revealed submucosal denudation, trachealization, and esophageal strictures (Figure 1B). Histopathological and immunohistochemical evaluation of biopsy specimens showed detachment of the squamous epithelium from the lamina propria, spongiosis associated with intraepithelial and subepithelial inflammatory infiltrates predominantly composed of CD3-positive T-cells (Figures 1C–F). There was no evidence of epidermoid metaplasia or dysplasia. The endoscopic and microscopic features were suggestive of a severe form of lichenoid esophagitis or esophageal lichen planus, fitting in the category of severe esophageal lichen planus (HP2) (5).

A subsequent dermatological evaluation found no evidence of lichen planus on the skin or mucous membranes, although mild eczema was noted on the neck and upper back, accompanied by elevated IgE levels and type I sensitization, suggesting atopic predisposition. The DIF microscopy did not detect IgG, IgA, IgM, C3 or fibrinogen deposits at the epithelial basement membrane, leading to a diagnosis consistent with lichenoid esophagitis.

In our search for possible causes, we carried out a comprehensive laboratory investigation, which revealed several abnormalities. These included normocytic anaemia (haemoglobin 10.7 g/dL, normal range > 13.5 g/dL), lymphocytopenia (0.68 Tsd/μl, normal range > 1.1 Tsd/μl), mild vitamin D deficiency (15.8 mg/mL, normal range > 20 mg/mL) and severe vitamin C deficiency (<0.4 mg/L, normal range > 4.6 mg/L), the latter mainly due to oesophageal malnutrition. Hepatitis B, C and cytomegalovirus infections were excluded. However, the presence of recurrent oral candidiasis, non-cardiac transient ischaemic attacks, Epstein–Barr virus (EBV) viremia (EBNA-1-IgA-CLIA 5.62 S/Co, PCR 1922/IU/ml) and IgG hypergammaglobulinemia (23.2 g/L, normal range 7,00–16,00 g/L) raised suspicion of underlying immunodeficiency. Subsequent blood tests confirmed active HIV infection with a high viral load (559,032 copies/ml) and a low CD4+ lymphocyte count (42/µl, normal range > 300/μl), categorised as C3. Eleven years ago the patient worked for 9 months each in Indonesia and Nigeria, which we hypothesize as the potential period of HIV infection. He had a one-time hospitalisation following an accident with loss of consciousness, but diagnostic or therapeutic regimen did not suggest any source of infection. Despite denials of known risk factors such as MSM, blood transfusion, hemophilia, or high-risk heterosexual contacts, the mode of infection remained undetermined.

A further histopathological workup of his esophageal lesions showed a nuclear HPV16 positivity of rare cells, while local EBV and HPV16 infections were excluded. Initiation of antiretroviral therapy (initially Bictegravir/Emtricitabin/Tenofoviralafenamid and then Dolutegravir and Lamivudine) markedly improved the esophageal mucosal condition, and further dilation of a 9 mm stricture significantly enhanced the patient’s food intake ability. One month after the start of antiretroviral therapy, the HIV viral load had significantly decreased (373 copies/ml), and the number of CD4+ cells had increased (127/µl). Histology of follow-up endoscopy four months later showed no evidence of the previously noted interface band-like lymphocytic infiltrate, indicating a substantial clinical improvement. During the first 12 months, the patient had a strong need to come to terms with the new diagnosis and stabilize both psychologically and socially.

## 3 Discussion

In this case report, we explored the clinical presentation of a patient with lichenoid esophagitis, absent of other lichen planus-associated mucocutaneous symptoms, who was concurrently diagnosed with HIV. This raises critical questions regarding the differential diagnosis of esophagitis-specifically, how to distinguish between lichenoid esophagitis and isolated esophageal lichen planus.

Lichen planus is an inflammatory disorder affecting squamous epithelia of the skin and mucous membranes. Its global prevalence is estimated between 0.5 to 2% (5). Oral lichen planus is the most common manifestation of mucosal lichen planus (3). It affects two thirds of patients with cutaneous lichen planus (8–10). Esophageal lichen planus on the other hand is described as rare, although the true prevalence is unknown (11). Interest in this disease has increased recently, but the data on its clinical manifestation is still incomplete and diagnostic management and treatment are partially standardized. Published case series and retrospective reviews show that most patients affected are middle-aged or elderly women, with a median age of 60–65 years. They usually report lichen planus in other locations and suffer from dysphagia (3, 12).

The diagnostic complexity is further heightened by the similarity of esophageal lichen planus symptoms to other forms of esophagitis such as gastroesophageal reflux disease (6). Unlike gastroesophageal reflux disease, esophageal lichen planus usually does not respond to proton pump inhibitor therapy and primarily affects the upper esophagus, as opposed to the lower third affected in gastroesophageal reflux disease (6). Eosinophilic esophagitis is another differential diagnosis of esophagitis (13). Diagnostic criteria for eosinophilic esophagitis encompass a typical clinical picture with trachealization (which can resemble esophageal lichen planus), more than ≥15/HPF eosinophil granulocytes and exclusion of differential diagnosis (14). The histologic aspect of the esophageal biopsies of our patient did not fulfil criteria of eosinophilic esophagitis since intraepithelial eosinophils were not increased and basal cell hyperplasia was absent. Epithelial detachment and spongiosis and apoptotic keratinozytes (civatte bodies), surrounded by intraepithelial T-cells were consistent with the morphologic pattern typically seen in esophageal lichen planus rather than in lymphocytic esophagitis (2).

Lymphocytic esophagitis, another rare esophageal condition, was first described by Rubio in 2006 (15). It is characterized by an increased presence of lymphocytes in the esophageal mucosa with rare or absent granulocytes (13, 15). Some authors such as Salaria et al. state that esophageal lichen planus and lichenoid esophagitis are subtypes of lymphocytic esophagitis, although the presence of degenerate keratinocytes is more indicative of esophageal lichen planus (2). But the diagnosis of lymphocytic esophagitis is controversial, with some physicians like Hussein et al. questioning the existence of a “new” disease entity and suggesting that lymphocytic esophagitis is probably a variable histologic manifestation of more common disorders (15).

In this case, the diagnostic puzzle gradually pointed to esophageal lichen planus, but the absence of extraesophageal manifestations raised the question of whether it was isolated esophageal lichen planus or lichenoid esophagitis. There is limited literature differentiating these entities. Salaria et al. use the term “lichenoid esophagitis pattern” when histology suggests esophageal lichen planus without DIF confirmation and no other symptoms of lichen planus are present (2). Both lichenoid esophagitis pattern and esophageal lichen planus predominantly affect females and share histopathological features, including lymphocytic band-like infiltrate at the interface between the squamous epithelium and the lamina propria, apoptotic keratinocytes, partial or complete detachment of the epithelium from the tunica propria, and dyskeratosis, making differentiation challenging (2, 6). However, Salaria et al. note that strictures are more frequently detected in esophageal lichen planus (2). According to Schauer et al. fibrinogen deposits can be detected in most cases of severe esophageal lichen planus using DIF microscopy (5).

Distinguishing between these diseases is important because they may be associated with different conditions or triggers and therefore be attributed to different pathogenic backgrounds. With regard to our case, fortunately the final diagnosis led to the detection of a previously unrecognized HIV infection. HIV infection on the other hand is known to be associated with various mucosal and gastrointestinal manifestations (16–18). A common condition is HIV-associated esophagitis, and patients with HIV have a higher risk of developing eosinophilic esophagitis (19). However, esophagitis in HIV patients can also be caused by various pathogens, including infections by Candida species, herpes simplex viruses, and cytomegalovirus (20, 21), resulting in mucosal lesions, erosions, and ulcers in the esophagus. These conditions cause symptoms such as dysphagia, pain, and difficulty swallowing, and the severity of pathological changes in the esophagus in HIV infections depends on the patient’s immune status and the presence of comorbidities (17–19).

According to historical data, patients with hepatitis C infection appear to have a higher risk of developing lichen planus, though some newer studies refute this (1, 22–24). There is, however, very little information on the association between HIV and lichen planus. There are few case reports of patients with HIV and lichen planus, most of whom suffered from mainly hypertrophic cutaneous lichen planus (25). There is even less data on the connection between HIV and lichenoid esophagitis or esophageal lichen planus. According to the review of Salaria et al. viral diseases such as hepatitis B, C or HIV were only seen in lichenoid esophagitis pattern and not in esophageal lichen planus, but the origin of the association is unclear (2).

The unique aspect of our case is the significant improvement observed after the initiation of antiretroviral therapy. This normalisation was seen in HIV viral load, ongoing but improved CD4+ lymphocyte count, and significant reduction of oesophageal inflammation. This adds an interesting dimension to our understanding of the interaction between HIV infection and oesophageal health.

In conclusion, the scarcity of literature on the association between HIV and lichenoid esophagitis underscores the need for future studies to explore this relationship further. The aim should therefore be to detect both diseases as early as possible to prevent stenosis and potential malignant transformation (2). With this case report we would like to raise the awareness for lichenoid esophagitis and their potential infectious associations. When treating patients with an esophagitis of unknown origin, even with no cutaneous symptoms, physicians should consider the differential diagnosis of esophageal lichen planus or lichenoid esophagitis.

## 4 Conclusion

In conclusion, this case underscores the diagnostic challenges of esophageal lichen planus and lichenoid esophagitis, particularly in the absence of mucocutaneous manifestations. The significant improvement following antiretroviral therapy suggests a link between HIV infection and esophageal inflammation. However, the pathomechanistic relationship between lichenoid esophagitis and HIV remains unclear. Future studies should explore this relationship further to enhance diagnostic and therapeutic strategies for similar patients. When dealing with esophagitis of unknown cause, it is important to consider both esophageal lichen planus and lichenoid esophagitis, especially after more common causes have been ruled out. Accurate and prompt identification is crucial to prevent progression to neoplasia and to identify potential comorbidities like HIV infection.

## Data Availability

The original contributions presented in the study are included in the article/supplementary material, further inquiries can be directed to the corresponding author.
